# Variable sensitivity multimaterial robotic e-skin combining electronic and ionic conductivity using electrical impedance tomography

**DOI:** 10.1038/s41598-023-47036-5

**Published:** 2023-11-15

**Authors:** Aleix Costa Cornellà, David Hardman, Leone Costi, Joost Brancart, Guy Van Assche, Fumiya Iida

**Affiliations:** 1 Physical Chemistry and Polymer Science, Vrije Universiteit Brussel, 1050 Brussels, Belgium; 2 Bio-Inspired Robotics Lab, University of Cambridge, Cambridge, CB2 1PZ UK

**Keywords:** Electronic devices, Sensors and biosensors

## Abstract

Electronic skins (e-skins) aim to replicate the capabilities of human skin by integrating electronic components and advanced materials into a flexible, thin, and stretchable substrate. Electrical impedance tomography (EIT) has recently been adopted in the area of e-skin thanks to its robustness and simplicity of fabrication compared to previous methods. However, the most common EIT configurations have limitations in terms of low sensitivities in areas far from the electrodes. Here we combine two piezoresistive materials with different conductivities and charge carriers, creating anisotropy in the sensitive part of the e-skin. The bottom layer consists of an ionically conducting hydrogel, while the top layer is a self-healing composite that conducts electrons through a percolating carbon black network. By changing the pattern of the top layer, the resulting distribution of currents in the e-skin can be tuned to locally adapt the sensitivity. This approach can be used to biomimetically adjust the sensitivities of different regions of the skin. It was demonstrated how the sensitivity increased by 500% and the localization error reduced by 40% compared to the homogeneous case, eliminating the lower sensitivity regions. This principle enables integrating the various sensing capabilities of our skins into complex 3D geometries. In addition, both layers of the developed e-skin have self-healing capabilities, showing no statistically significant difference in localization performance before the damage and after healing. The self-healing bilayer e-skin could recover full sensing capabilities after healing of severe damage.

## Introduction

Human’s ability to feel a wide range of tactile inputs, slight temperature differences and pain, is made possible by the complex network of sensory receptors and nerves found on our skin. The skin not only acts as a sensor but it is also a barrier against external agents. In addition, it possesses unique mechanical properties and the ability to heal physical damage^1^. In the pursuit of replicating such capabilities in robotic systems, the field of electronic skin has emerged^2–4^. Electronic skin (e-skin) has become an important research topic in the areas of soft robotics^3^, machine-human interaction^5^, and tracking of movement or physiological activity^6,7^.

A biomimetic e-skin for soft robotics must be stretchable, cover large areas, and have high spatiotemporal resolution^3^. To achieve these requirements, active sensor matrices are commonly used^8^. In recent years, substantial advances have increased the number of sensors and the complexity of e-skin^8^. However, such approaches complicate the fabrication process, especially at large scales, and their fragility and repair difficulties are still issues that need to be tackled. A simplified version of such systems consists of the use of a matrix formed of conductive perpendicular lines, where each intersection becomes a strain sensor^9,10^. Despite their simple working principle, they still present repair and fragility problems, which hinder their use at a large scale.

An alternative solution is electrical impedance tomography (EIT): a technique that allows the estimation of the internal conductivity distribution of a body. This distribution is obtained by injecting a known current through the skin and measuring the potential from electrodes attached to its boundary. Since its introduction in the early 1980s^11^, the main use for EIT has been found in medical devices^12^. However, since its first use for tactile sensors in 2007 by Nagakubo et al.^13^ electrical impedance tomography has received a lot of interest in large-scale e-skins^14–18^ because it has the potential to solve these fabrication, robustness, and repairability problems^19^.

Despite its advantages, EIT sensors have several drawbacks. The most well-known drawback concerns the quality of the analytically reconstructed image, due to the simplifications and assumptions used to solve the ill-posed inverse problem^16,20^. To address this, reconstruction methods based on machine learning have been recently introduced^15,21,22^. Another critical problem arises from the distribution of electrons in the sensor. In typical configurations where the electrodes are placed at the boundary of the geometry, the areas far from these electrodes are notably less sensitive than the regions near the boundary^16^. Also, the geometries that can be achieved with such configurations are very limited^23^. Introducing electrodes inside the measuring area reduces this problem^15,16,23,24^, but interferes with the skin’s tactile interaction mechanics. Additionally, the fabrication process becomes more complicated or even impossible for complex geometries. By keeping electrodes only at the skin’s periphery, fabrication is kept straightforward and the key interaction areas remain unaffected by electrode integration.

**Figure 1:**
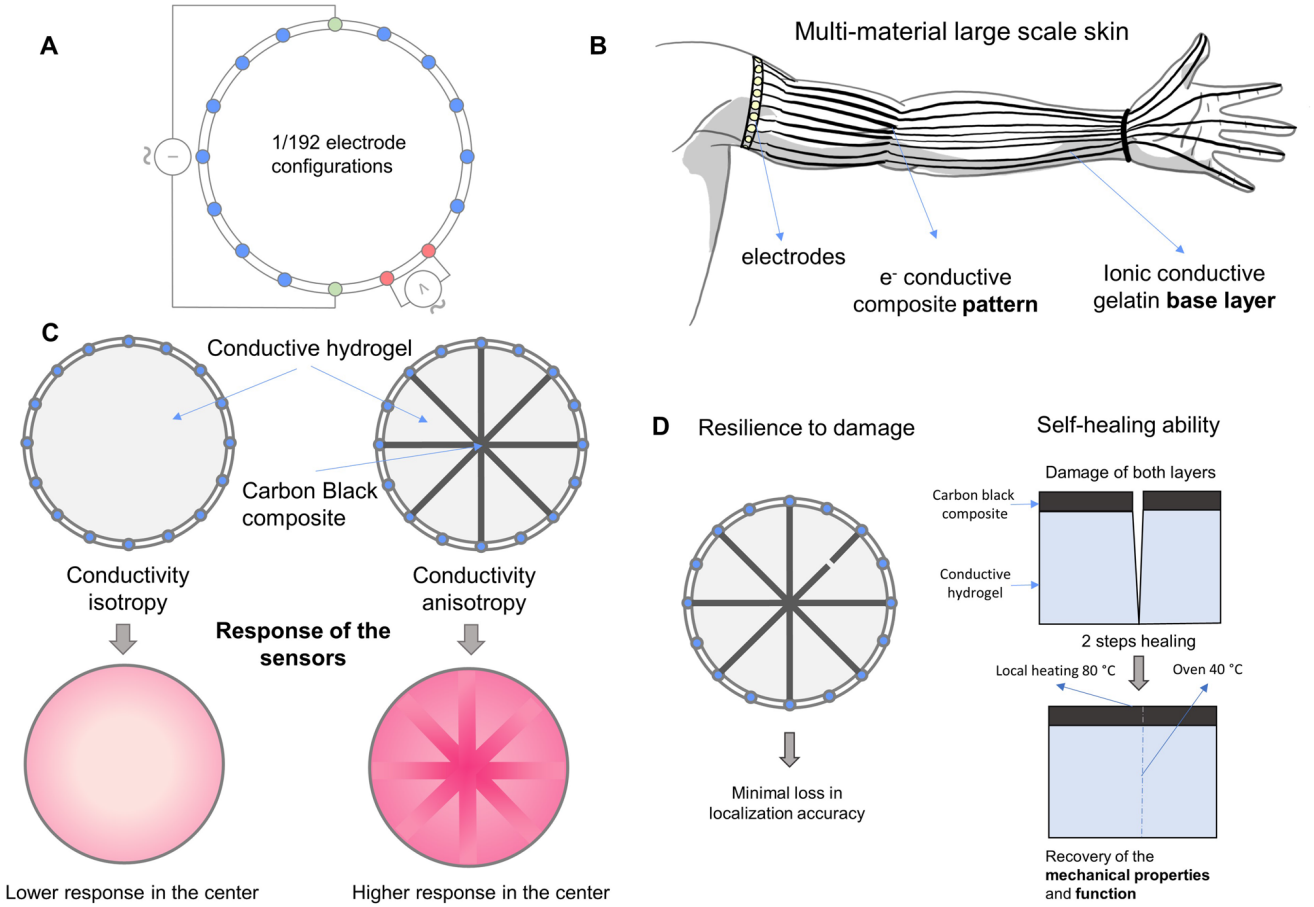
(**A**) One configuration of the EIT electrodes: AC current is injected between two opposite (green) electrodes, while the voltage is measured between two adjacent (red) electrodes. (**B**) The multi-layer e-skin enables sensitivity tuning of areas far from the electrodes, such as the fingertips of a sensorzied arm. (**C**) Our multi-layer design (right) can increase the sensitivity of areas which would have low sensitivity for homogeneous skins (left). (**D**) In addition to the intrinsic resilience to damage of the sensors based on EIT, the developed e-skin can self-heal large physical damages.The self-healing process is shown in the cross section scheme of the multi-layer e-skin..

In biological skin, sensitivity to touch varies depending on the area of the body^25,26^. Such differences are key to humans for tactile discrimination, protective response, social interaction, and well-being^27^. Inspired by this, we present a multi-layer e-skin design approach, which allows the sensitivities of different areas to be tuned. This method can be used to address the problem of lower sensitivity in areas far from the electrodes when EIT is used. The skin consists of two layers: the base layer composed of a self-healing gelatin hydrogel, and the top layer made with an electronically conductive carbon black-filled self-healing material. By patterning the top layer (Fig. 1C), we can tune the e-skin’s sensitivity to specific applications via the resulting changes in current density. Since the two materials use different conductive mechanisms, this introduces anisotropic behaviors which we characterize for use in EIT-based e-skins (Fig. 1A). In particular, we consider how this framework could be used for more complex geometries, where areas far from the electrodes typically face little sensitivity. By taking advantage of the anisotropic behaviors of our composite material, areas far from the electrodes (such as the fingertips in Fig. 1B) could be tuned to high sensitivities, whilst the whole area remains responsive. In addition, both components of the skin are self-healing (Fig. 1D), adding durability to the already high robustness of EIT e-skins^15^. We aim for this approach to find its use in robotic skins with complex geometries, where having electrodes all over the area is not possible. Additionally, in future iterations, we aim to add other capabilities to these robotic skins, such as temperature sensing, which could be incorporated in this EIT e-skins via a multi-layer stacking^28^.

## Results

### Multilayer e-skin with anisotropic conductivity

**Figure 2:**
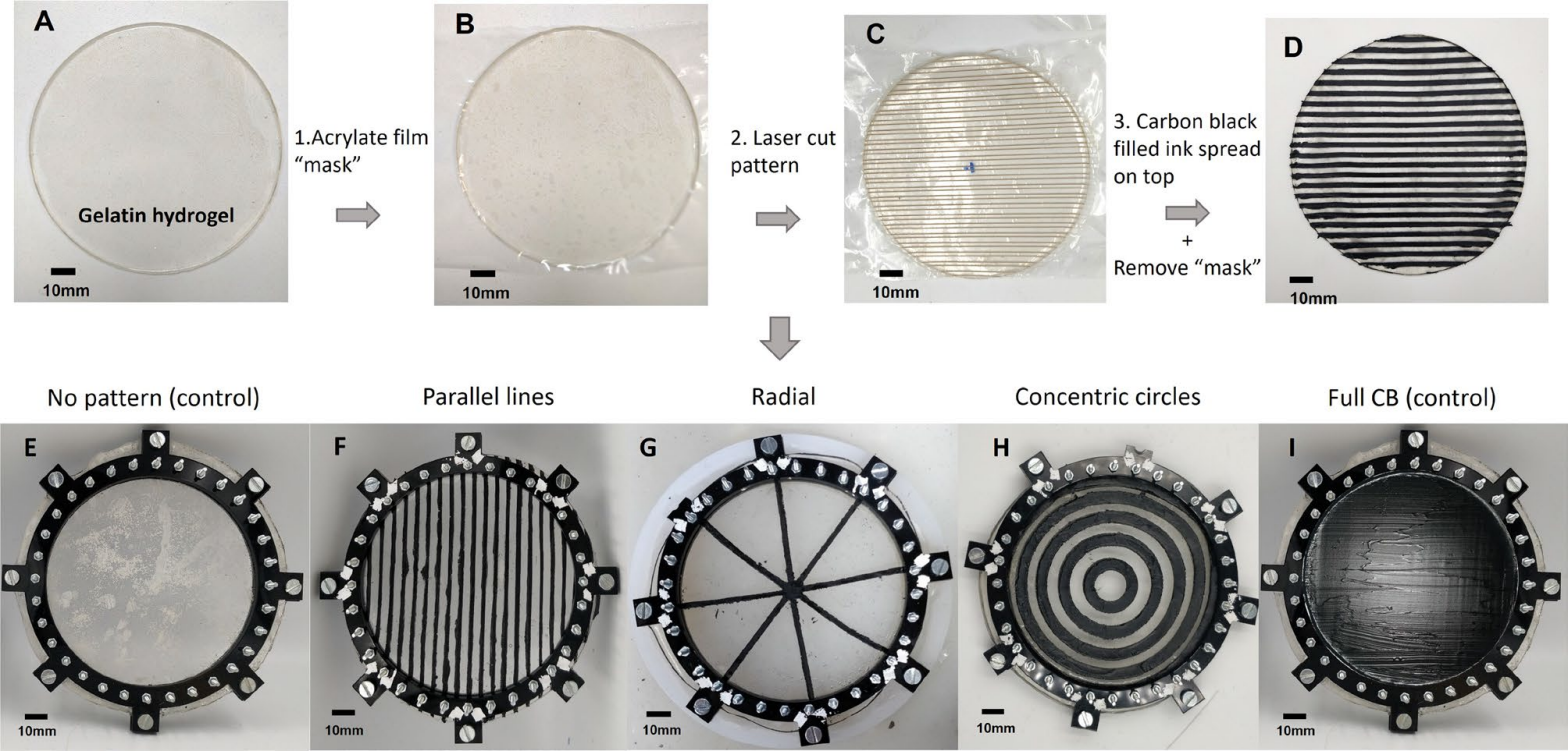
(**A**–**D**) Fabrication procedure of the electronically conductive pattern on top of the gelatin hydrogel. (**E**–**I**) Picture of the different patterns and control samples tested in this work. (**E**) No pattern, (**F**) Parallel lines, (**G**) Radial, (**H**) Concentric circles, and (**I**) Full CB.

The e-skin is formed by two active layers stacked together (Fig. 2). Both layers are conductive, but their conductivity mechanisms are different. The base layer is made of gelatin hydrogel and has ionic conductivity, while the top layer is a self-healing elastomer filled with carbon black (CB) that conducts electrons through the percolating CB network. Since the term ’electrical conductivity’ encompassed the charge transport through any type of carrier, ions and electrons included, the terminology ’electronic conductivity’ is used to differentiate the charge transport via electrons from ions^29,30^. By combining the two, anisotropic behaviors are introduced to both the baseline resistance and the skin’s piezoresistive response. Gelatin hydrogels show a positive increase in resistance when they are subjected to a strain due to a decrease in the cross-sectional area, which reduces the flow of current^31,32^, while CB-filled elastomers in the first 20% strain show a negative response^33^. Carbon black was used instead of other electrically conductive fillers, such as silver nanowires or liquid metal, because the resistance of the CB thin film deposited on top of the gelatin has a resistance in the same order of magnitude as the gelatin hydrogel. We speculate this match is important for our approach to have part of the current flowing through the two materials rather than having one material carrying all the current. To explore the potential of their combined anisotropy for EIT e-skins, three patterns were tested with different geometries of the electronically conductive top layer (Fig. 2F–H), designed to significantly alter the paths of least resistance compared to the homogeneous case. These are compared against homogeneous samples with no top layer (Fig. 2E) and a complete top layer (Fig. 2I). Details of their fabrication are presented in the “Methods” section. These are patterns that traditional reconstruction algorithms struggle to detect at the resolutions under consideration (Supplementary Figure S1), and we therefore adopt a data-driven approach.

**Figure 3:**
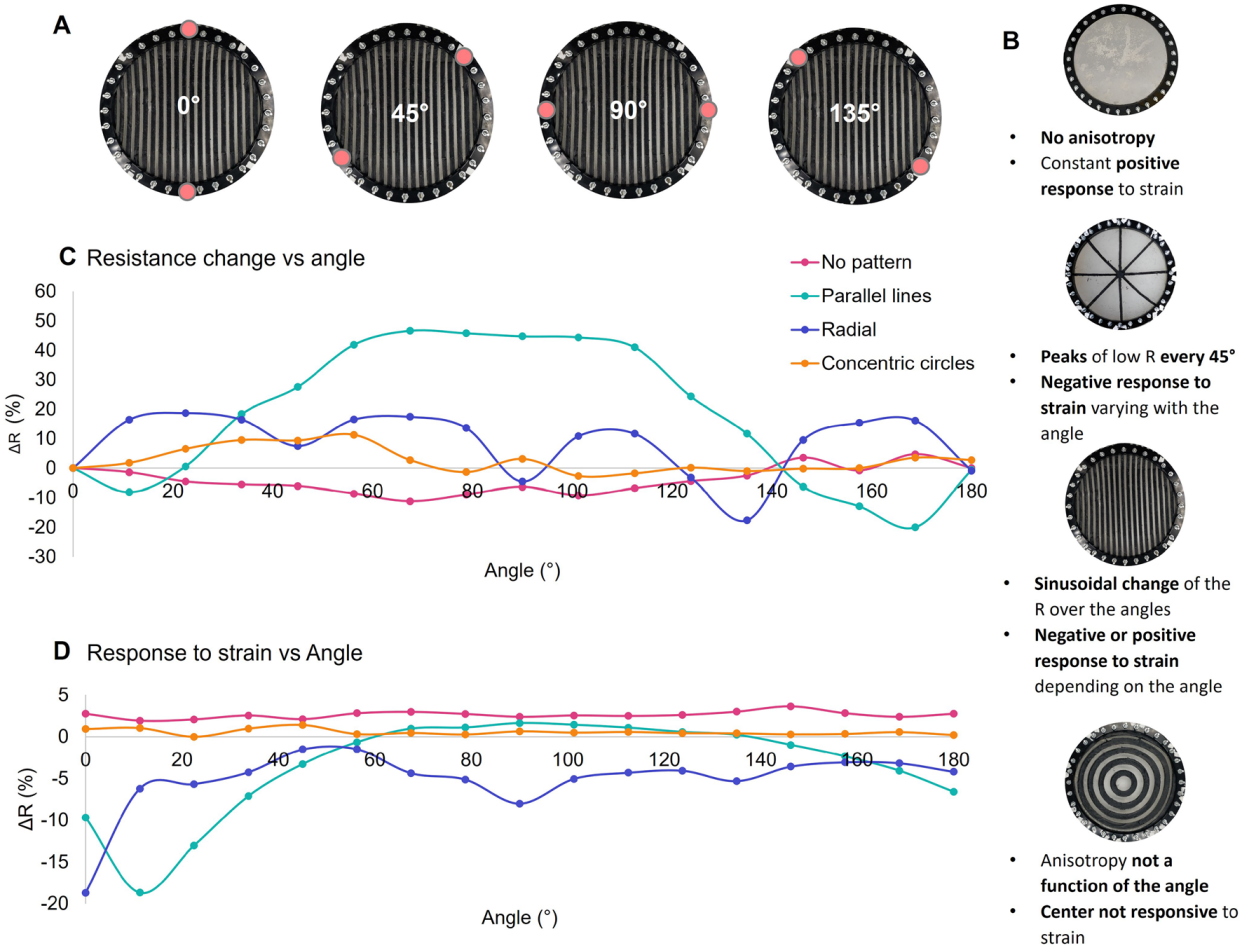
Anisotropy results in resistance and response to strain versus the angle of measurement. (**A**) Schematic representation of the electrodes used to measure the resistance at different angles. (**B**) Summary of the change of resistance and response to strain versus angle for the different patterns. (**C**) Change of baseline resistance with angle, compared to the resistance at 0$^{\circ }$. (**D**) Angle-dependent responses to a 1 cm depth touch in the center of the patterns.

In order to assess its robustness, the mechanical properties of the materials alone, and combined in the multi-material structure, were tested (Supplementary Figure S10). It was observed that both materials can withstand deformations over 100% and that the multilayer structure outperforms the mechanical properties of the gelatin alone. After assessing the material’s mechanical performance, we tested whether these patterns lead to anisotropy in the piezoresistive response, measuring resistance across the diameter at a variety of angles (Fig. 3A). In Fig. 3C, the plotted values correspond to the change in resistance compared to the resistance measured at an angle of 0$^{\circ }$. Similarly, to measure how the piezoresistive response changes with angle, the resistance for each angle is measured before and during a manual press of 1 cm depth in the middle of the skin. The difference between these values as a function of the angle is reported in Fig. 3D.

As a control, a sample without a pattern on top was tested. This sample is isotropic, there is little change in resistance with angle (Fig. 3C). Some difference is observed, caused by inhomogeneities in the fabrication process, such as bubbles, or slight thickness variations. The response to a strain is positive and angle-insensitive, which is in agreement with what is expected from an isotropic ionically conductive hydrogel. When a pattern of electrically conductive parallel lines is applied, we observe anisotropy and because of the geometry of the parallel lines, this anisotropy is angle dependent. The resistance changes follow a sinusoidal wave with a peak of maximum resistance when the resistance is measured perpendicular to the parallel lines. The response to strain is also angle dependent. The response is negative when the resistance measurements are done parallel to the conductive lines and it is positive when the resistance measurements are done perpendicular to the lines, smoothly transitioning from one regime to the other in the intermediate angles. At the intersection between the two regimes, there is an angle where the resistance does not respond to strain. In the case of our sample, this angle can be found at an angle between 60$^{\circ }$ and 70$^{\circ }$. In the radial geometry, it is possible to observe a decrease in resistance when the measurement is aligned with the electrically conductive paths. The response to strain is always negative which means that the strain behavior in the center is mostly controlled by the conductive lines. Finally, the concentric circles pattern has anisotropy, but this anisotropy is not angle dependent. Therefore, the resistance doesn’t change with the angle. More interestingly, the response to strain in the center is almost zero. This is due to the fact that most electrical current takes the path of least resistance which we have tuned to avoid this region. A summary of the effects of the different patterns in the resistance and response to strain can be seen in Fig. 3B.

### Touch localization based on EIT

**Figure 4:**
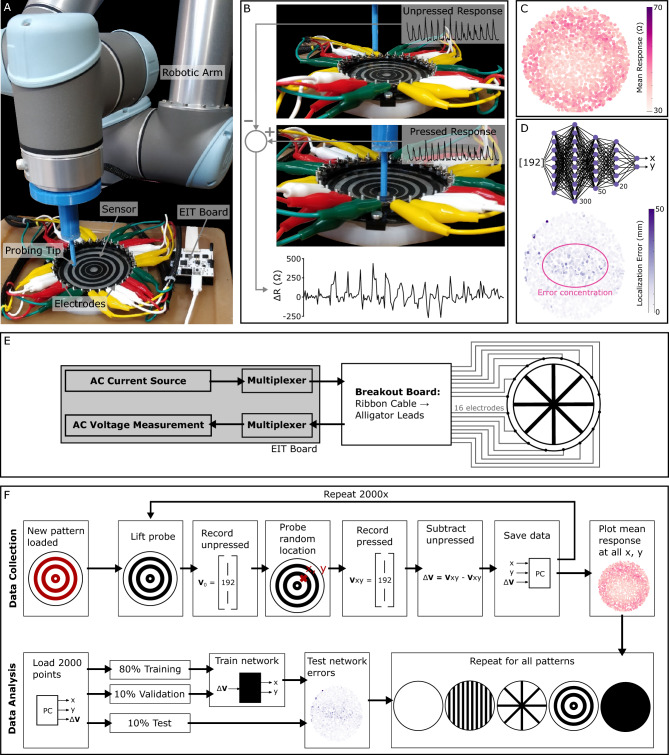
(**A**) The automated testing setup: a robotic arm probes random locations on a sensorized skin. (**B**) Signal extraction: for each press, the difference between the pressed and unpressed states is recorded for 192 electrode configurations. (**C**) Response maps show the average response over the 192 configurations at each of the 2000 probed points. (**D**) Error maps show the localization error of a feedforward neural network trained to predict the coordinates of the press. (**E**) Schematic of the electrical setup. (**F**) Flowchart of the entire process.

Having confirmed that the different patterns cause basic changes to the composite material’s electrical properties, the effect on the tetrapolar tomography measurements was assessed, taken from the circumferential electrodes. Both the CB-filled elastomer and the hydrogel are conductive and deform under pressure, altering the e-skin’s conductivity distribution. The detection of these changes can be done using electrical impedance tomography (EIT) hardware. In EIT, an alternating current is injected between two of the electrodes, and the voltage is measured between adjacent electrodes. 16 electrodes were used (Fig. 4E), resulting in 192 tetrapolar electrode configurations. The response is recorded for a series of randomly located presses performed by a robotically operated 5 mm probe (Fig. 4A). Each response is obtained by subtracting the 192 signals of the unpressed state from the 192 signals of the pressed state (Fig. 4B). 2000 uniformly distributed random presses are performed on each e-skin.

To evaluate the performance of each pattern in the context of tunable e-skins, we focus on two aspects: how sensitive the e-skin is to deformations at particular locations (Fig. 4C), and how accurately the deformations can be localized by a trained neural network (Fig. 4D). A flowchart of the testing and evaluation procedure can be seen in (Fig. 4F).

#### Sensitivity: mapping the responses of the e-skin

**Figure 5:**
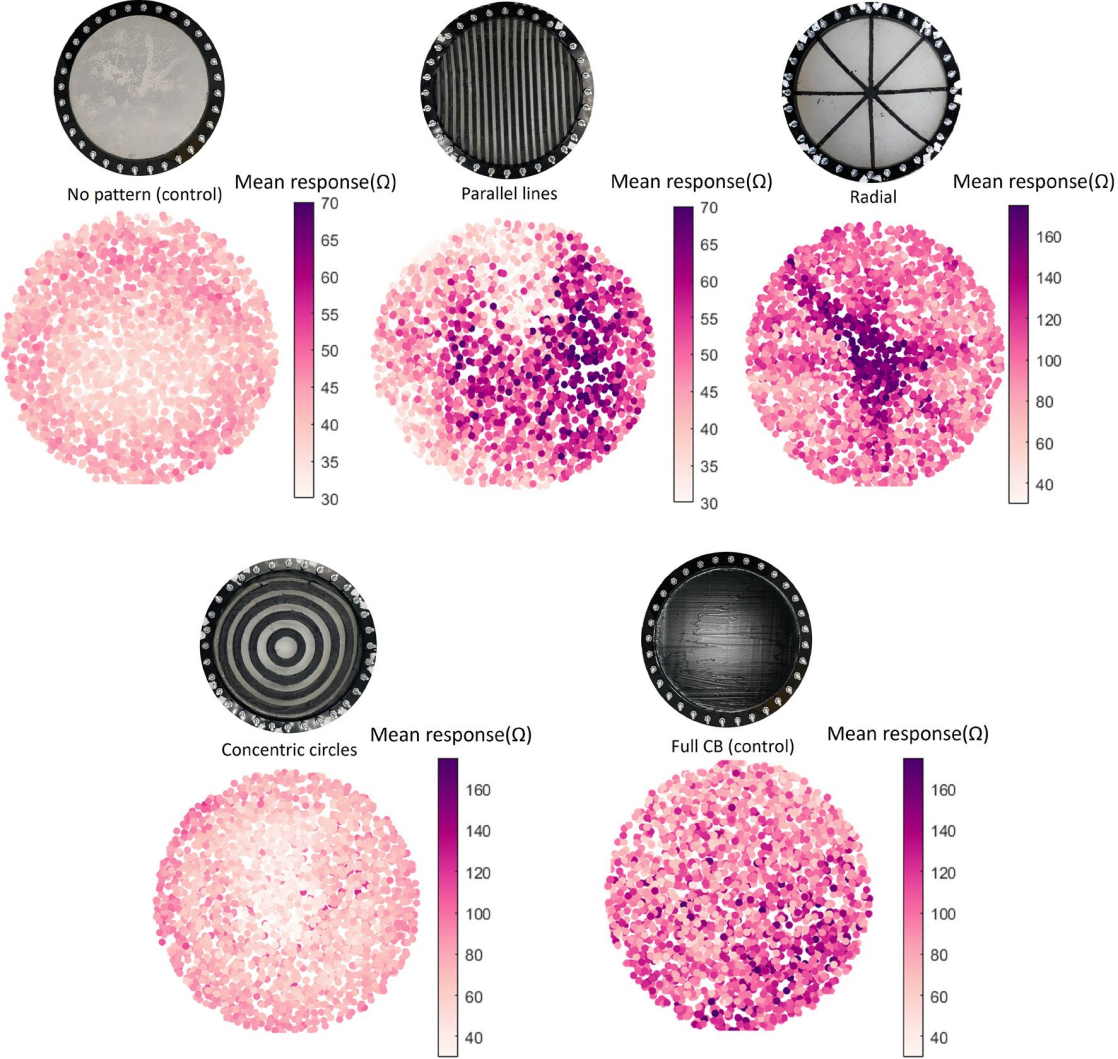
Response maps for the different control and test samples. Each map is made by plotting the average of the absolute values of the response over the 192 possible electrode configurations for each touch position.

First, it was evaluated how the patterned composites introduce different regions of sensitivity into the EIT skins. To do so, a ‘response maps’ was used for each sample (Fig. 5). These maps are made by plotting the average of the 192 absolute channel values (specific channels are plotted in Supplementary Figure S2) at each the 2000 pressed locations: higher values suggest a greater mean response from the channels, corresponding to higher sensitivities of the region. It is known in literature that sensitivity far from the electrodes is problematic^16^. Here we confirm that by showing that the sensitivity of the homogeneous hydrogel e-skin’s center is lower than the response near the electrodes. This is greatly improved in the patterns of the parallel lines and the radial geometry, where the response is higher. The center of the e-skin in the sample with the radial geometry has an average response 6 times higher than the e-skin with no pattern on it, from an average response of 30 $\Omega$ to an average response of 180 $\Omega$ (Fig. 5). As expected, the central area of the sample with concentric circles becomes even less sensitive than the sample with no pattern on it. This is because the current that passes through the center is greatly reduced, as it mostly passes through the less resistive paths made of CB-filled elastomer, hence its response is also reduced. With these maps, we demonstrate that using electrically conductive patterns on top of the gelatin hydrogel increases the responsiveness of the e-skin and that we can tune which areas are more responsive by changing the pattern used. We expect that by increasing the number of radial lines, or concentric circles, the overall mean response would increase. This increase in overall response is especially beneficial for geometries with bigger areas with low response. Hence, the bigger the areas of low response the higher the density of electrically conductive lines should be in order to keep the accuracy high. Some differences can be observed between the two sides of the parallel lines and the different lines of the radial pattern. We attribute this differences to imperfections during the fabrication. Differences in thickness can affect the conductivity of different lines of the pattern, which affects the responsiveness.

#### Accuracy: deep neural network for touch localization

Having seen that the anisotropic patterns lead to different sensitivities of the skins, their effect on localization predictions was explored, training a feed-forward neural network (described in the “Methods” section) to predict the *x* & *y* coordinates of a probe, by way of the skin’s 192 sensor responses. By using a data-driven approach, we not only learn to localize tactile stimuli from the fabricated pattern anisotropies, but also benefit from fluctuations in the material properties and hysteresis, tuning our network to each specific sensor to maximize accuracy.

**Figure 6:**
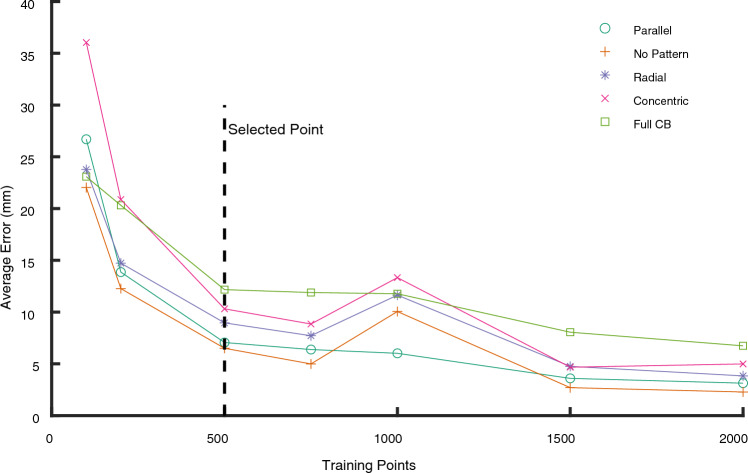
Average localization error versus number of training points for a neural network trained on each of the patterned skins. Each To minimize the amount of real-world data required, 500 training points are selected for subsequent tests.

To determine the number of known responses required for satisfactory training, Fig. 6 compares the test set’s average error between predicted and actual probe tip locations for a network trained on a random selection of 100, 200, 500, 750, 1000, 1500, or 2000 presses. Given the expense of collecting this real-world data, approximately 12h to collect 2000 presses, the minimum number of training points that can be used without significantly raising the error was determined. 500 training points were selected for subsequent tests and figures, since this marks the start of the plateau in Fig. 6. Though smaller errors are possible using more data points, it was determined that 500 data points are sufficient to explore the tunable sensitivities.

**Figure 7:**
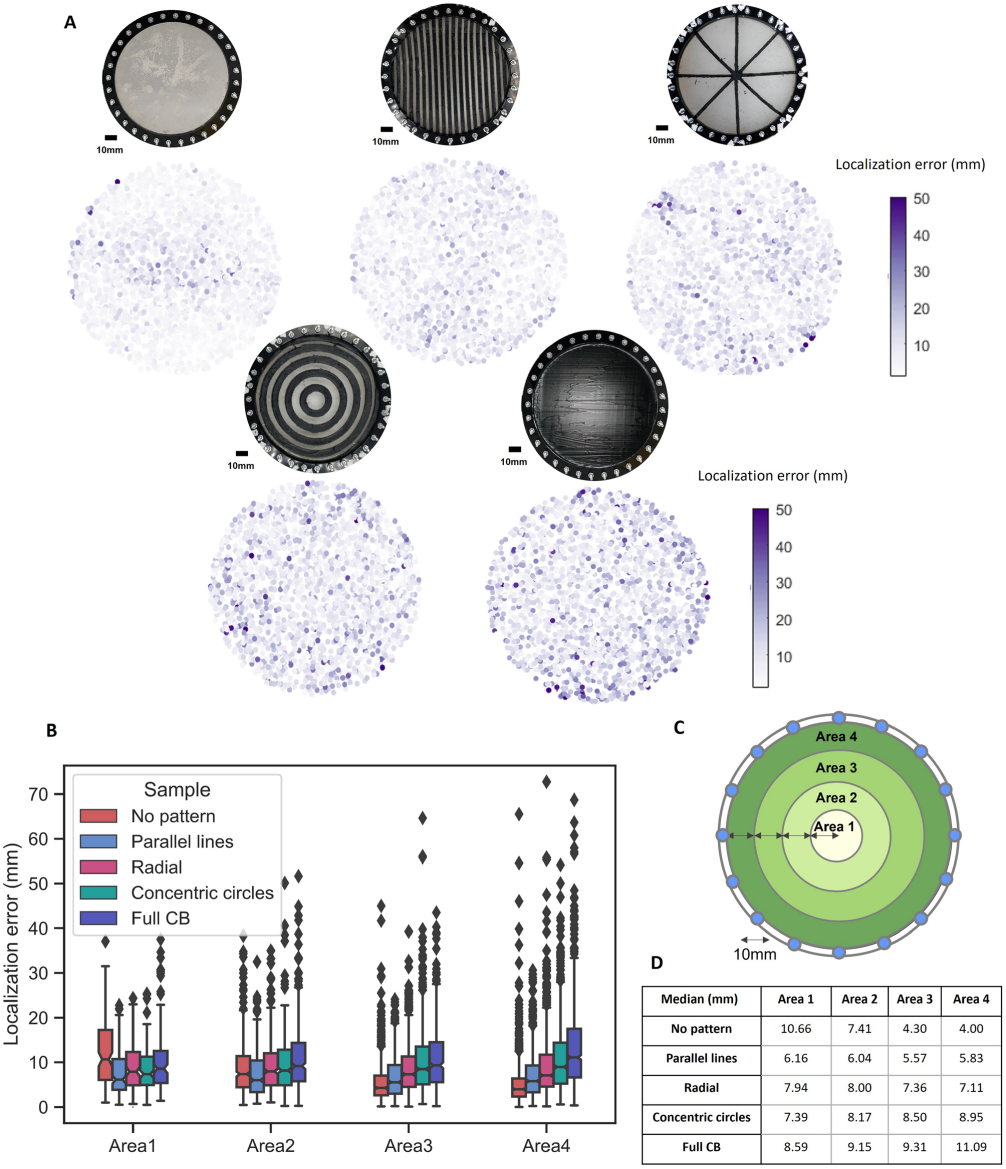
(**A**) Localization error distribution depending on the touch position for the different test and control samples. (**B**) Boxplot of the results of the localization error depending on the touch area (**C**). The skin is split into four concentric areas. (**D**) Table summarizing the median of the localization error for the different samples and areas.

Clear differences in the errors of the different skins are apparent in Fig. 6. The fully covered skin always has higher errors than the skin without pattern due to the higher hysteresis of the CB-filled elastomer^34^. Most of the patterned skins fall between these two extremes. The distribution of these errors was evaluated based on the location on the skin (Fig. 7A). The skin without pattern clearly outperforms the full CB coverage, with smaller errors over the entire area. However, we now see how the composite patterned skins are able to shift the areas of highest and lowest error: whilst the ‘no pattern’ skin has the highest errors in its center (as expected from Fig. 5), this is one of the lowest areas of error for the radially, concentrically, and parallel patterned skins. Similarly, the parallel line pattern results in vertical bands of lower error at the sides of the skin compared to the concentric and homogeneous cases.

To quantify these shifts in localization accuracy, Fig. 7B divide the skins into 4 concentric areas (displayed in Fig. 7C), over which the network’s test set errors are plotted. Thorough statistical analyses are presented in Supplementary Figures S3–S8. In the regions closest to the electrodes (Areas 3 & 4), the material itself has the biggest effect: no pattern and full CB have the lowest and highest errors respectively, with the patterned cases falling between these. However, it is in the center (Area 1) where the greatest effect of the patterns can be seen: the no pattern case now shows the highest median error of 10.66 mm. The parallel lines show the most consistency in errors between the three areas (varying by just 0.33 mm), indicating that similar patterns might be selected when uniform accuracy is desired for complex geometries. In this way, our results validate the concept of sensitivity tuning—patterns can be designed to give the greatest localization accuracy in areas of high tactile interest—such as the fingertips—at the expense of other lesser-used areas, such as the forearm. Here, the skin’s center is the location at which this benefit is most apparent; due to the opposite electrode injection pattern, the homogeneous skin’s center has current passing across it during all measurements. As the principle is extended to more complex 3D geometries, this is not necessarily the case for critical areas, and we expect to see significant benefits by diverting the path of least resistance to reach these areas in future investigations. This enables electrodes to be physically distanced from the most sensitive areas, preventing them from interfering with physical interactions. The ‘optimum pattern’ is application dependent, and formalized design processes should be developed to propose patterns for a variety of reward functions. In simulation, such optimization relies on an accurate models of the material behaviors, so physical iterative approaches could instead be employed, using Bayesian optimization to avoid the reality gap.

### Self-healing and resilience to damage

**Figure 8:**
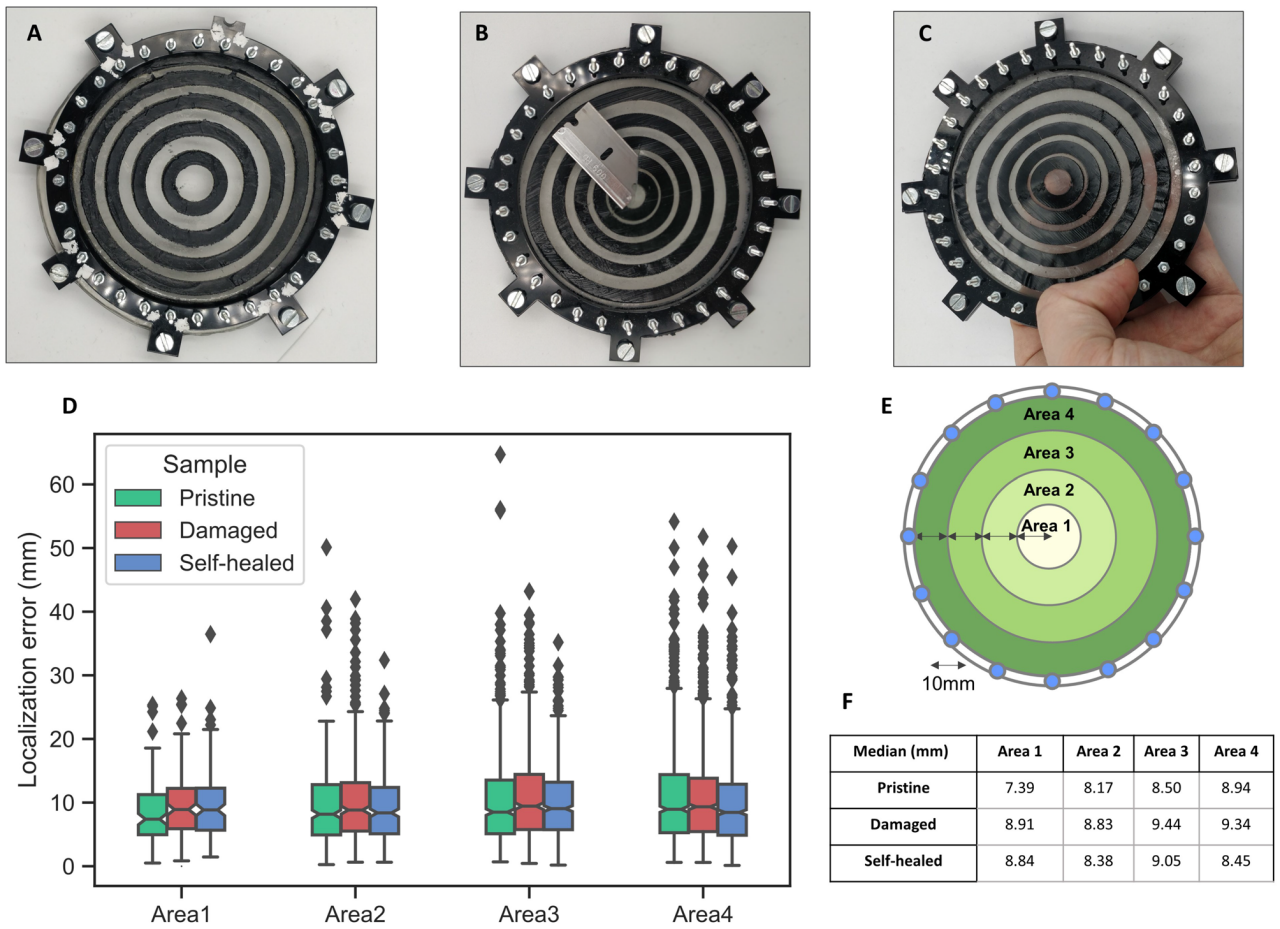
Pictures of the pristine concentric circles sample (**A**), Damaged (**B**), and self-healed (**C**). (**D**) Boxplot of the localization error of the samples at the three stages of the self-healing procedure at different areas. (**E**) The skin is split into four concentric areas (**F**) Table summarizing the median of the localization error for the different states and areas.

EIT-based tactile sensors are relatively resilient to physical damage^15^, which our design furthers by using only self-healing materials. The two materials have previously been shown to heal after damage^34–36^: while the gelatin hydrogel can partially heal at room temperature, or totally by slightly warming it up at 40 $^{\circ }$C, the CB filled elastomer requires a temperature of around 80 $^{\circ }$C. This mismatch in temperatures means that self-healing becomes a two-step process. First, the damaged parts are placed together and gently heated in the oven at 40 $^{\circ }$C for 30 min. Subsequently, the damaged elastomer parts are locally heated to achieve self-healing. In order to test this, a 2 cm cut is inflicted in the center of the e-skin (Fig. 8A–C). This confirms the resilience to damage to the EIT sensors, as the localization of the error while the damage is not repaired is still under 10 mm using 500 touches for the training (Fig. 8D), with an increase in the localization error compared to the pristine sample (Fig. 8F). After the self-healing process, the physical integrity of the sensor is recovered (Fig. 8C), and the localization error decreases compared to the damaged case (Fig. 8F).

In order to assess if the results obtained are statistically relevant, we perform a Mann–Whitney U test comparing the three samples (Pristine, Damaged, and Self-healed, Supplementary Figure S9). We find that the pristine versus damaged and the self-healed versus damaged are statistically different, with the error of the damaged being higher, and that there is no statistical difference between the localization error between the pristine and the self-healed sample.

## Conclusions

In this work, we have introduced a general strategy to tune the local sensitivity of electronic skins (e-skins) based on Electrical Impedance Tomography (EIT) without the need for a complex system of electrodes. This strategy is based on the combination of two materials with different conductivities and charge carriers, introducing anisotropy to the resistance and piezoresistive responses. A circular e-skin was fabricated out of a self-healing ion-conducting hydrogel with or without a pattern of carbon black-based self-healing composite on top. The sensitivities of different areas of the e-skin can be tuned by changing the geometry of the top layer to increase the response in areas far from the electrodes. It was confirmed that this tuneability in sensitivity improves the accuracy of trained network touch localization. The biggest improvement was observed at the center of the circular skin, with a reduction of the localization error of 40% and an increase in sensitivity by 500% compared to the skin with a uniform top layer. Additionally, the e-skins can recover from large physical damage through a simple two-step healing procedure, showing no statistically significant difference in localization performance before the damage and after healing.

In this work, we provided a preliminary demonstration of the working principle of these e-skins at a material level. Future research will focus on developing the tools and analysis to further process the obtained signals for full deformation reconstructions, minimizing the localization errors, and enabling the fabrication and application of large-scale e-skins for complex geometries. In particular, the integration of a formalized pattern design process into the manufacturing stages would facilitate optimal sensorization of these bodies. Additionally, we will also work on the development of reconstruction algorithms that can work with the multi-material structures developed in this work.

## Methods

### Materials

Succinic anhydride (SA) and castor oil (CO; 164 mg KOH/g) were obtained from Sigma-Aldrich. 4-tert-Butylcatechol was used as a radical inhibitor and was obtained by Sigma-Aldrich. Furfuryl glycidyl ether (FGE) was obtained from BLD Pharmatech GmbH, Kaiserlauten, Germany. BMI-689 was obtained from Designer Molecules (*Willow Creek, San Diego*). Carbon black was obtained from Thermo Scientific (*Waltham, Massachusetts, United States*). All products were used as received. For the hydrogel, the glycerol, gelatin powder, and citric acid were obtained from Fisher Scientific, MM ingredients, and Fisher Scientific respectively.

### Functionalization of the castor oil with furan groups

The Castor oil is functionalized with furan groups via a two-step one-pot synthesis^35^. The first step is a ring-opening esterification to castor oil using succinic anhydride. The reaction is done in a round flask under a $N_2$ atmosphere and reflux, without solvent, and magnetically stirred. A molar proportion of 1:1 between the hydroxyl groups form the castor oil and succinic anhydride is used. The reaction is done at 125 $^{\circ }$C for 24h. The product of the synthesis (sCO) is used in the next step without any further purification. The second step of the synthesis is an epoxy-carboxylic acid reaction between FGE and sCO. The reaction was done in the same round flask as in the first step, $N_2$ atmosphere and reflux, without solvent, and magnetically stirred. A molar proportion of 1:1 between the hydroxyl groups form the castor oil and the FGE. The reaction is done at 100 $^{\circ }$C overnight. The product of the synthesis (FsCO) is used in the next step without any further purification.

### Fabrication of the multi-material anisotropic e-skin

The multi-material e-skin is prepared in two steps. First, the conductive gelatin base layer is prepared by mixing together gelatin (pork, 240–260 bloom), glycerol, water, citric acid monohydrate and table salt (NaCl) in a weight percent proportion of 1:1.5:2.5:0.2:0.075 respectively. All the components are mixed and heated up at 50$^{\circ }$C for a minimum of 4h and cast into a laser-cut circular polymethylmethacrylate (PMMA) mold with a diameter of 12 mm. The material is left to dry at room temperature for at least 2 days it partially dries until it reaches an equilibrium with the environmental humidity. The method to prepare the gelatin base layer is based on the work of Hardman et al.^36^. Once this equilibrium is reached, 4 layers of a 0.1 mm acrylic low-tack protection film are stuck on the surface of the gelatin. Subsequently, the desired pattern is laser-cut (HPC Laser Ltd, LS 6090 PRO), with a speed of 20 and a power of 17%. Then, the film corresponding to the design’s negative part is peeled off, hence exposing the gelatin. In parallel, a conductive ink paste is prepared by mixing using an ARM-310 mixer (Thinky), Carbon black powder, FsCO, BMI-689, and 4-tert-Butylcatechol with a composition of 0.25:0.67:0.33:0.0033 of the respective components. Once the pattern of the gelatin is exposed and the conductive ink is prepared, an homogenous layer of the ink paste is spread on top of the gelatin. Afterward, the remaining film is removed and the deposited conductive pattern is left to cure at room temperature for at least 48h.

### Experimental setup

Voltage measurements are collected using 16 electrodes (M2 stainless steel bolts) arranged uniformly in a 120 mm diameter circle around a laser-cut PMMA frame with an inner diameter of 110 mm. The e-skin is sandwiched between two of these laser-cut PMMA frames and pressed together to ensure electrical contact. The frame is placed on top of a 20 mm of Ecoflex 00–30 layer (fabricated by casting into a 3D printed mold) to mimic a soft body and to ensure that the deformation caused by contact with the skin is localized near the touch location. The 16 electrodes are wired to a commercially available multichannel Spectra electrical impedance measurement device (Minds Eye Biomedical). For each measured state, the board uses a combination of 25 kHz opposite electrode constant driving currents to yield a set of 192 adjacent electrode voltage measurements. Throughout the experiment, the skin is pressed using a Universal Robots UR5 robotic arm, equipped with a 3D printed polylactic acid (PLA) probe of 5 mm diameter and 40 mm length. It presses at random locations within the 110 mm diameter of the skin. 2000 random touches are recorded for each pattern, which takes approximately 12 hours. Before each press, a full set of 192 measurements is recorded. Then the probe descends and the press is held in position until another full set of 192 measurements is taken.

### Neural networks

Feedforward neural networks are used to predict the location of the presses, trained using real-world data with a 80:10:10 % training/validation/test data split. In Figs. 7 and 8, 500 datapoints are first used, selected randomly from the 2500 presses. All additional points are then incorporated as test data for these plots, giving an effective 16:2:82 % split. Based on results from the authors’ previous work^14^, a fixed architecture is used throughout, implemented using MATLAB’s Deep Learning Toolbox. An input layer of size 192 is followed by three hidden layers with 300, 50, & 20 nodes respectively, and a 3-node regression output (*x*, *y*, & depth, which is held constant at 18.8 mm in this study). A tanh activation layer follows each hidden layer. Training (stochastic gradient descent with momentum) begins with a learn rate of 0.05, which falls by 1 % every 500 epochs, using minibatches of size 500. Training continues for 5000 epochs. Each input is taken to be the difference in response between the pressed and unpressed states in each of the 192 channels.

### Self-healing procedure

The e-skin is removed from the PMMA frames and a 2 cm cut is inflicted in the center of the e-skin with a scalpel. The freshly cut surfaces are put in contact and gently heated in the oven at 40 $^{\circ }$C for 30 min. Subsequently, the damaged electronically conductive elastomer parts are locally heated with a soldering iron at 80 $^{\circ }$ C for 30 s to achieve self-healing. The samples were left 24h at ambient conditions to stabilize before they were reassembled into the frame and tested.

### Stress–strain curves

The mechanical testing was performed on a TA Instruments DMA Q800 at ambient temperature. Stress–strain tensile tests and cyclic stress–strain tests were performed at room temperature using a film tension clamp. Rectangular specimens with a thickness of 1.25 mm and 5 mm width were clamped with a distance between clamps of 5 mm and strained at a rate of 60% /min.

### Supplementary Information

Supplementary Figures.

## Data Availability

The code and datasets generated and/or analysed during the current study are available in: https://github.com/DSHardman/Variable-Sensitivity-E-Skins.
