# Supplementary material for: Variable sensitivity multimaterial robotic e-skin combining electronic and ionic conductivity using electrical impedance tomography
Source: Sci Rep. 2023 Nov 15;13:20004. doi: 10.1038/s41598-023-47036-5 (PMC10651849; doi:10.1038/s41598-023-47036-5)
Supplement: Supplementary file 1 — Supplementary Figures. [file 41598_2023_47036_MOESM1_ESM.pdf]

Supplementary information

Variable Sensitivity Multimaterial Robotic E-Skin Combining Electronic and Ionic Conductivity using Electrical Impedance Tomography

Aleix Costa Cornellà[1,2], David Hardman[1], Leone Costi[2], Joost Brancart[1], Guy Van Assche[1], and Fumiya Iida[2*]
[1]Vrije Universiteit Brussel, Physical Chemistry and Polymer Science, Brussels, 1050, Belgium
[2]University of Cambridge, Bio-Inspired Robotics Lab, Cambridge, CB2 1PZ, United Kingdom
*fi224@cam.ac.uk

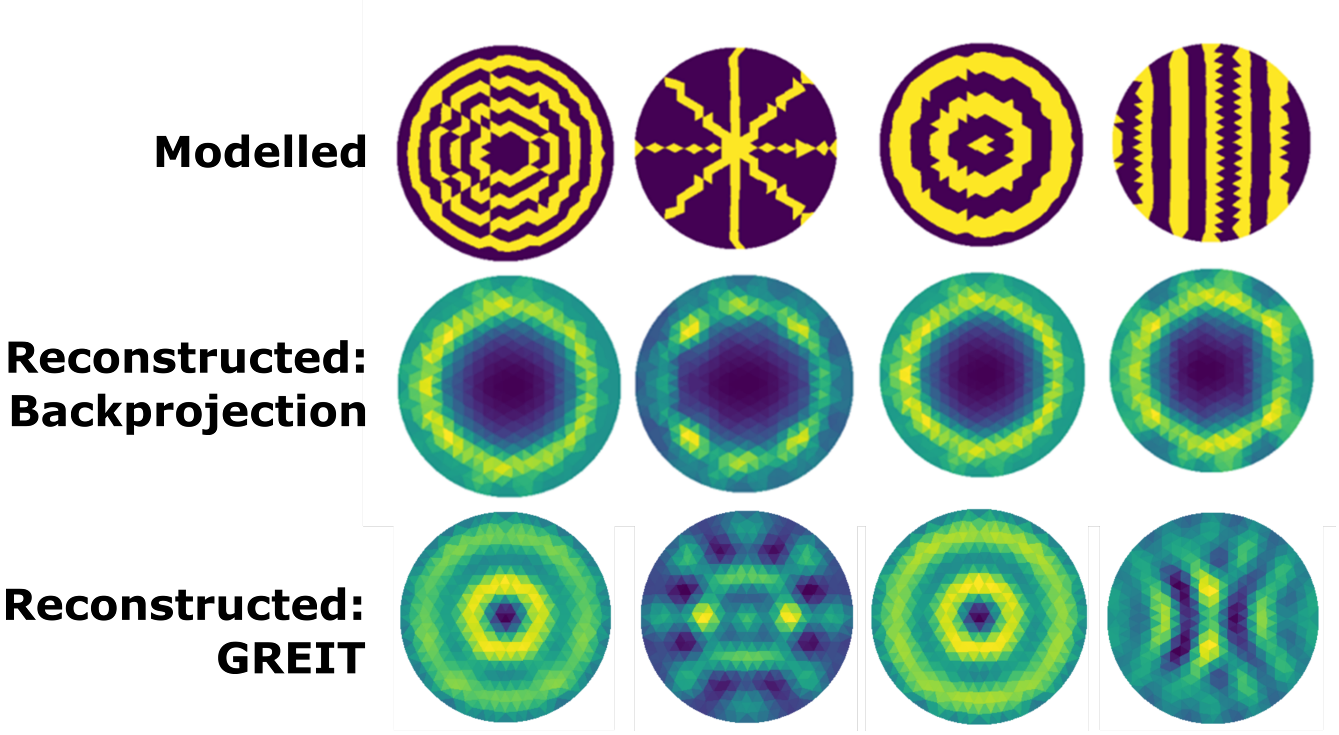

Figure S1. Analytic reconstructions of the patterns considered in this work, passing binary conductivity maps through forward and inverse solvers implemented in pyEIT. Very little evidence of the patterns is visible using single-step backprojection, whilst these are much more apparent with GREIT. Still, details are lost, and there is little to distinguish between the wide and thin concentric patterns.

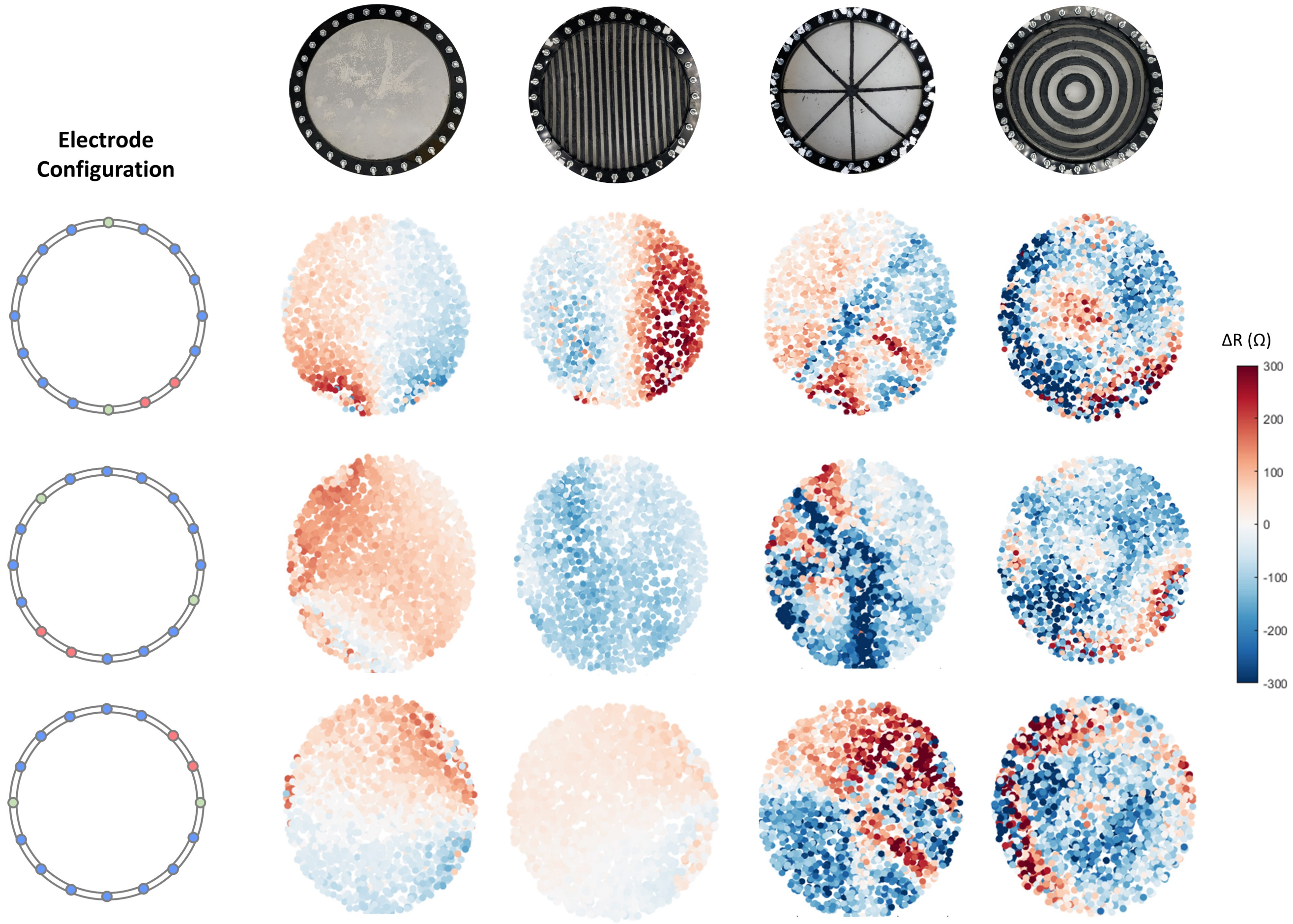

Figure S2. Response maps for the different control and test samples for three specific electrode configurations. Each response map is made by plotting the response of that specific electrode configuration at each touch position.

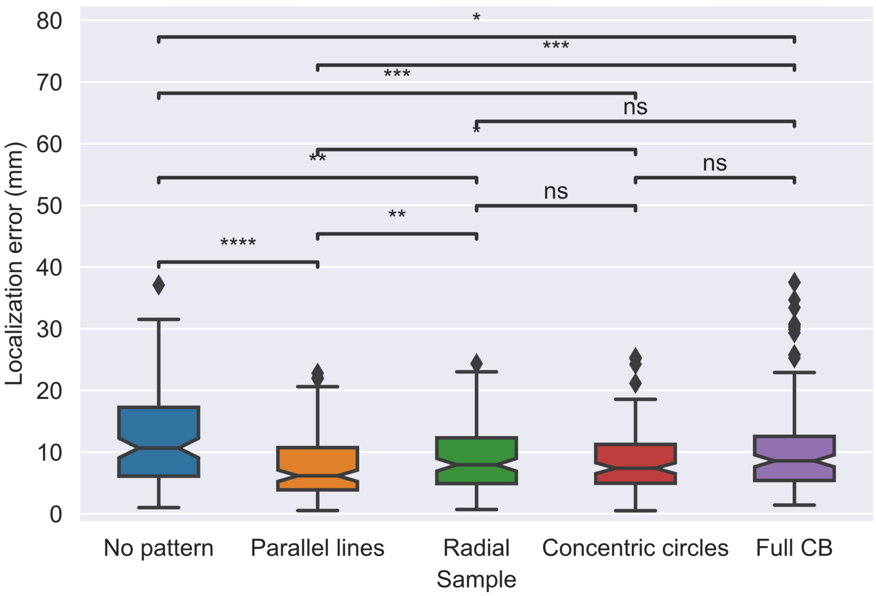

p-value annotation legend:
ns: p <= 1.00e+00
*: 1.00e-02 < p <= 5.00e-02
**: 1.00e-03 < p <= 1.00e-02
***: 1.00e-04 < p <= 1.00e-03
****: p <= 1.00e-04

No pattern vs. Parallel lines: P_val:2.101e-07 U_stat=1.132e+04
Parallel lines vs. Radial: P_val:6.908e-03 U_stat=6.974e+03
Radial vs. Concentric circles: P_val:3.898e-01 U_stat=8.568e+03
Concentric circles vs. Full CB: P_val:1.031e-01 U_stat=7.339e+03
No pattern vs. Radial: P_val:1.611e-03 U_stat=9.611e+03
Parallel lines vs. Concentric circles: P_val:3.231e-02 U_stat=7.200e+03
Radial vs. Full CB: P_val:4.016e-01 U_stat=7.939e+03
No pattern vs. Concentric circles: P_val:1.403e-04 U_stat=9.837e+03
Parallel lines vs. Full CB: P_val:4.166e-04 U_stat=6.683e+03
No pattern vs. Full CB: P_val:3.132e-02 U_stat=9.312e+03

Figure S3. Localization error boxplot at the Area 1 for the different patterns and control samples. The annotations on top of the boxplot correspond to the results of the Mann-Whitney U test between each possible pair. A summary of the results of the test can be found at the bottom right of the figure.

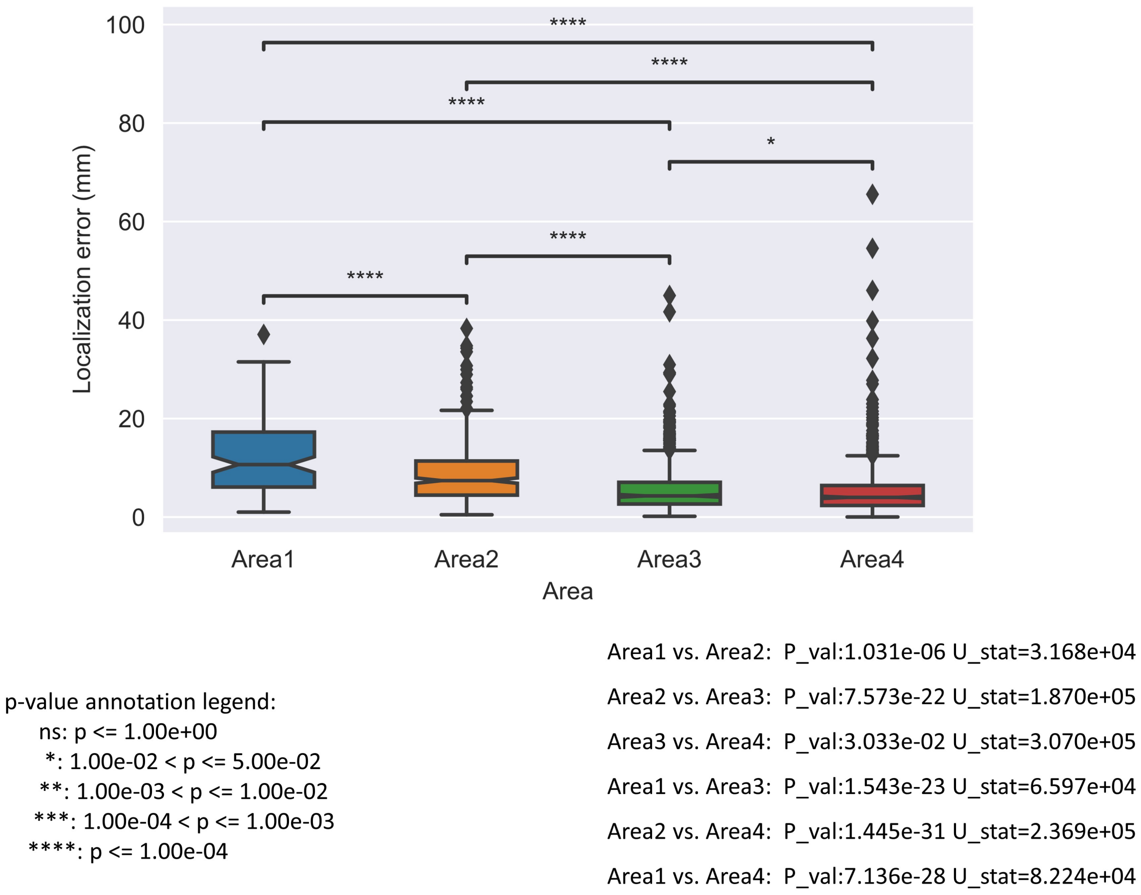

Figure S4. Localization error boxplot of the sample with no pattern on top for the different areas of the e-skin surface. The annotations on top of the boxplot correspond to the results of the Mann-Whitney U test between each possible pair. A summary of the results of the test can be found at the bottom right of the figure.

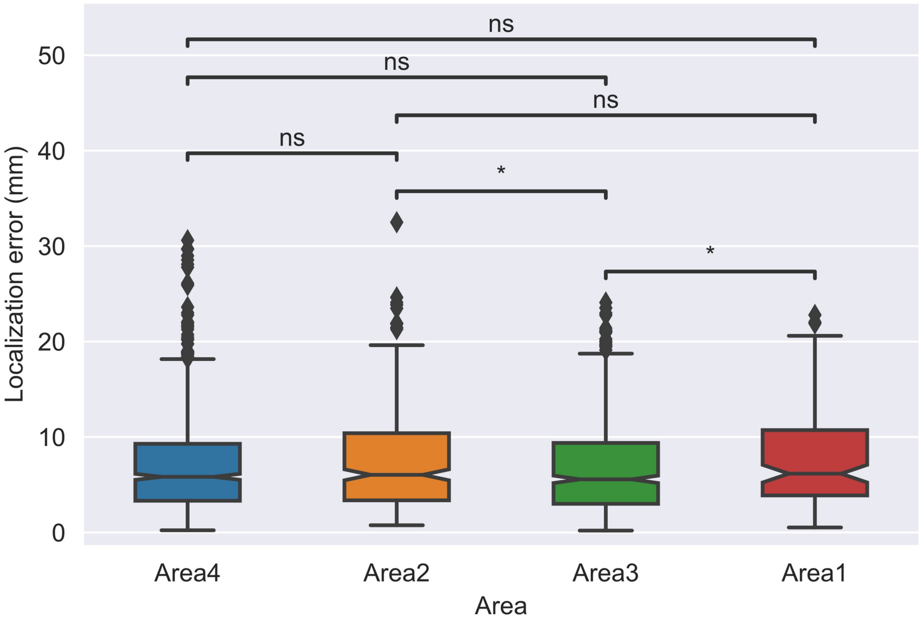

p-value annotation legend:
ns: p <= 1.00e+00
*: 1.00e-02 < p <= 5.00e-02
**: 1.00e-03 < p <= 1.00e-02
***: 1.00e-04 < p <= 1.00e-03
****: p <= 1.00e-04

Area3 vs. Area1: P_val:4.717e-02 U_stat=3.948e+04

Area2 vs. Area3: P_val:4.701e-02 U_stat=1.294e+05

Area4 vs. Area2: P_val:3.340e-01 U_stat=1.580e+05

Area2 vs. Area1: P_val:5.465e-01 U_stat=2.390e+04

Area4 vs. Area3: P_val:1.887e-01 U_stat=3.040e+05

Area4 vs. Area1: P_val:1.984e-01 U_stat=5.608e+04

Figure S5. Localization error boxplot of the sample with electrically conductive parallel lines on top for the different areas of the e-skin surface. The annotations on top of the boxplot correspond to the results of the Mann-Whitney U test between each possible pair. A summary of the results of the test can be found at the bottom right of the figure.

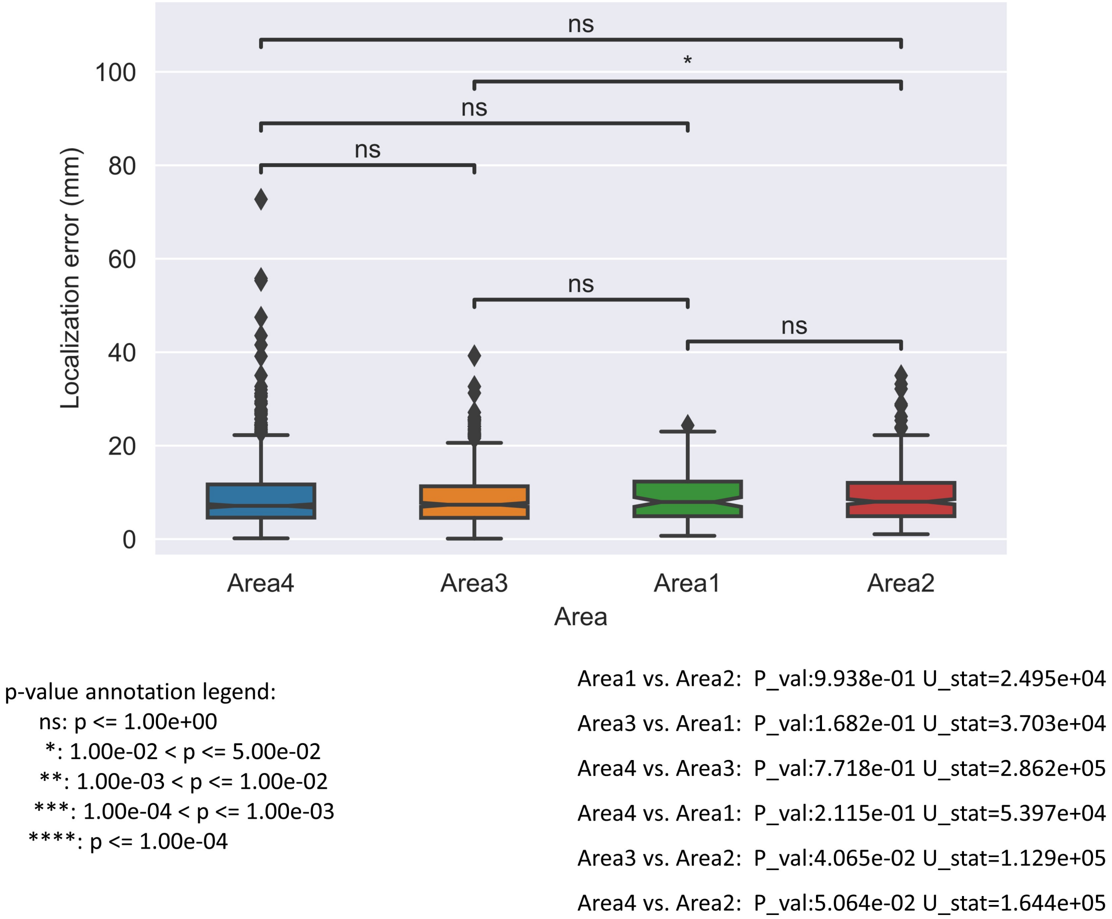

Figure S6. Localization error boxplot of the sample with electrically conductive radial lines on top for the different areas of the e-skin surface. The annotations on top of the boxplot correspond to the results of the Mann-Whitney U test between each possible pair. A summary of the results of the test can be found at the bottom right of the figure.

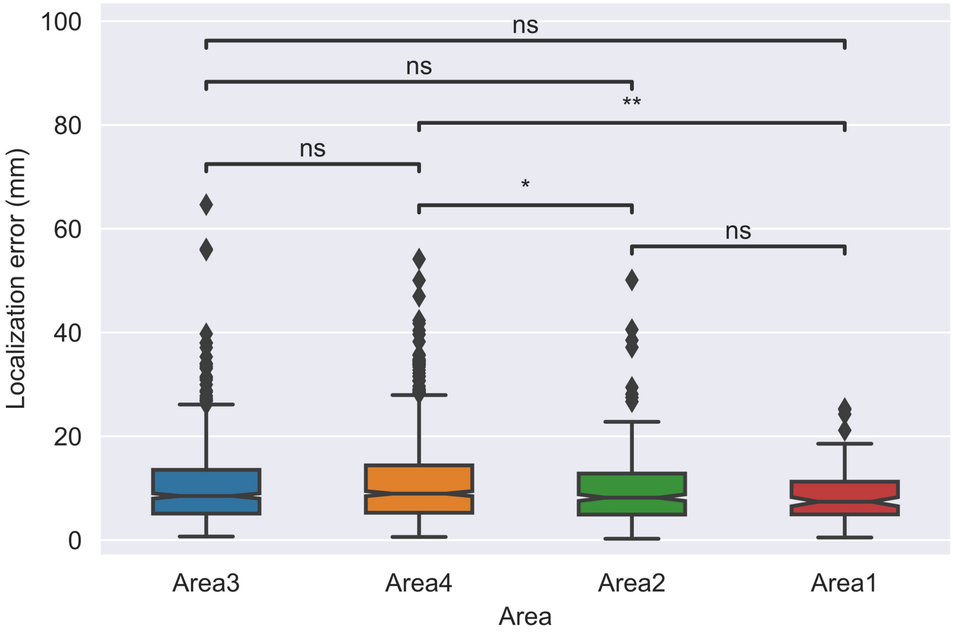

p-value annotation legend:
ns: p <= 1.00e+00
*: 1.00e-02 < p <= 5.00e-02
**: 1.00e-03 < p <= 1.00e-02
***: 1.00e-04 < p <= 1.00e-03
****: p <= 1.00e-04

Area2 vs. Area1: P_val:2.779e-01 U_stat=2.515e+04

Area4 vs. Area2: P_val:1.176e-02 U_stat=1.764e+05

Area3 vs. Area4: P_val:1.034e-01 U_stat=2.818e+05

Area4 vs. Area1: P_val:5.115e-03 U_stat=6.276e+04

Area3 vs. Area2: P_val:2.917e-01 U_stat=1.337e+05

Area3 vs. Area1: P_val:6.906e-02 U_stat=4.762e+04

Figure S7. Localization error boxplot of the sample with electrically conductive concentric circles on top for the different areas of the e-skin surface. The annotations on top of the boxplot correspond to the results of the Mann-Whitney U test between each possible pair. A summary of the results of the test can be found at the bottom right of the figure.

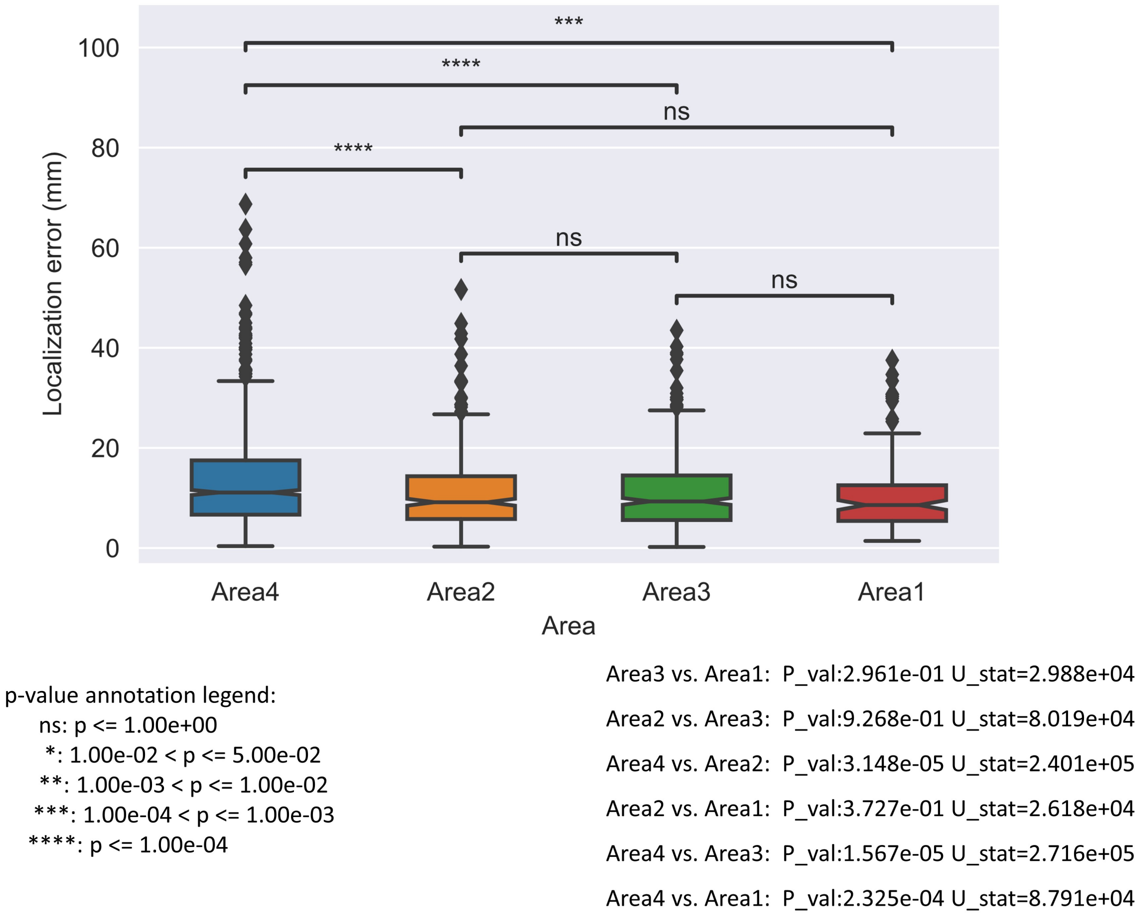

Figure S8. Localization error boxplot of the sample with a full layer of electrically conductive elastomer on the top of the hydrogel base layer for the different areas of the e-skin surface. The annotations on top of the boxplot correspond to the results of the Mann-Whitney U test between each possible pair. A summary of the results of the test can be found at the bottom right of the figure.

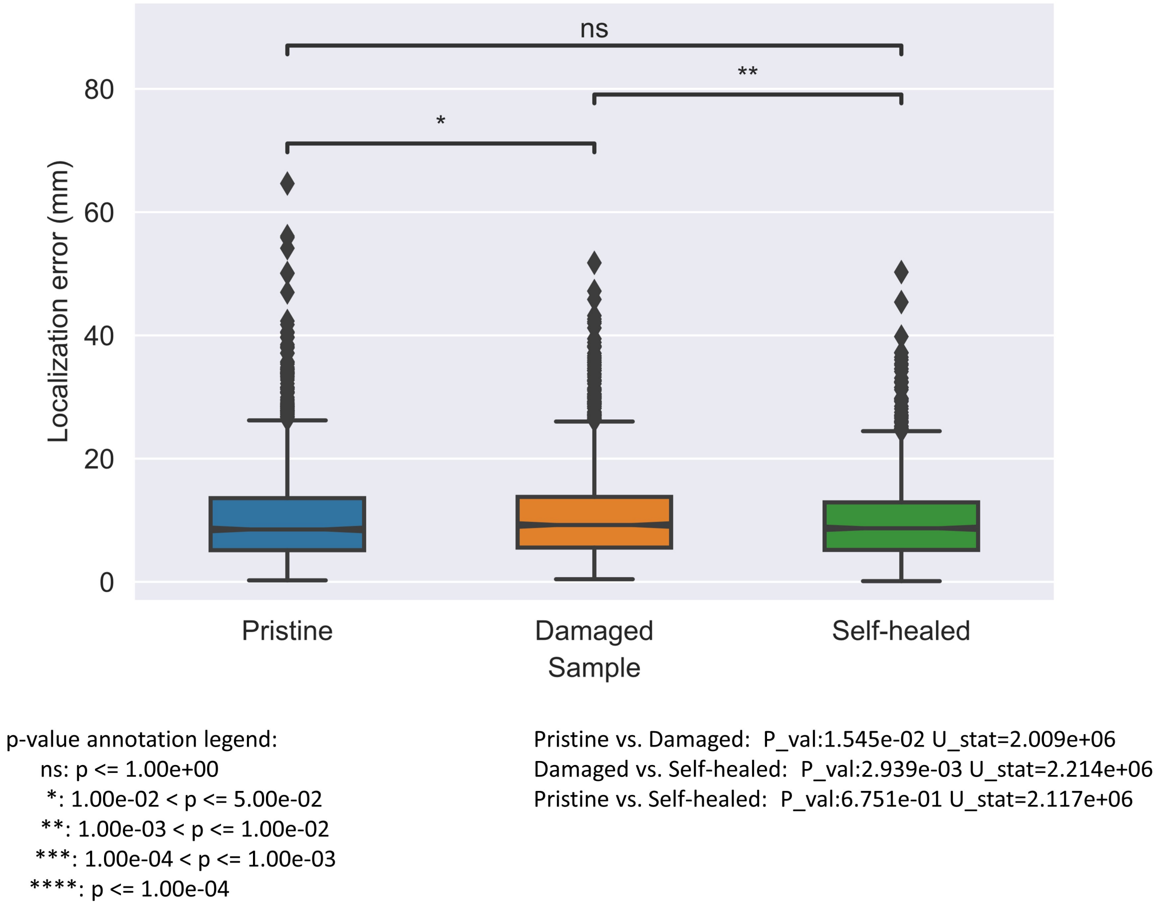

Figure S9. Localization error box of the samples at the three stages of the self-healing procedure. The annotations on top of the boxplot correspond to the results of the Mann-Whitney U test between each possible pair. A summary of the results of the test can be found at the bottom right of the figure.

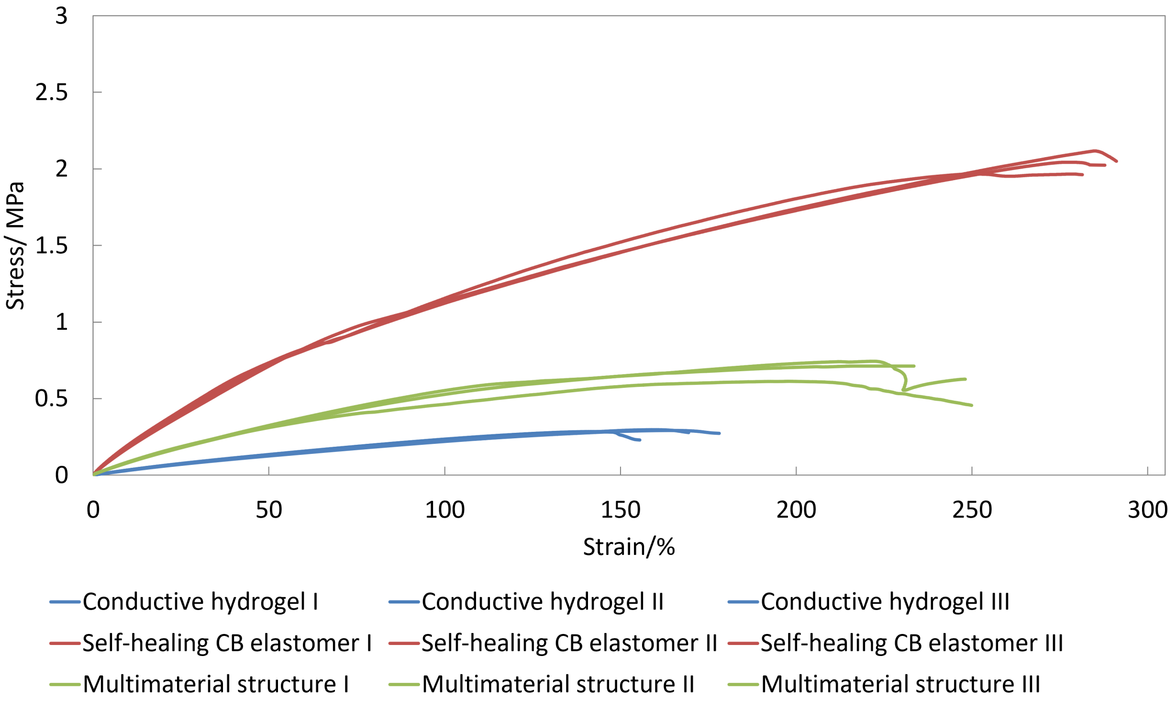

Conductive hydrogel

Mean			STDEV	
Stain at fracture	167.6667	%	11.39927	%
Stress at fracture	0.2909	MPa	0.00608	MPa
Young's modulus	0.346667	MPa	0.011547	MPa

Self-healing CB elastomer

Mean			STDEV	
Stain at fracture	286.7667	%	143.4399	%
Stress at fracture	2.042	MPa	1.02286	MPa
Young's modulus	2.8	MPa	0.2	MPa

Multimaterial structure

Mean			STDEV	
Stain at fracture	243.8333	%	122.1376	%
Stress at fracture	0.681267	MPa	0.347166	MPa
Young's modulus	1.5	MPa	0.264575	MPa

Figure S10. Stress-strain curves for the conductive hydrogel, the self-healing elastomer filled with carbon black, and the multimaterial stucture of the thin coating of arbon black filled elastomer on top of the conductive hydrogel. A summary of the mechanical properties can be found in the tables
